# Supplementary material for: Perceived Racial Discrimination, Psychological Distress, and Suicidal Behavior in Adolescence: Secondary Analysis of Cross-Sectional Data from a Statewide Youth Survey
Source: Healthcare (Basel). 2024 May 14;12(10):1011. doi: 10.3390/healthcare12101011 (PMC11121279; doi:10.3390/healthcare12101011)

Supplementary Materials

Perceived racial discrimination, psychological distress, and suicidal behavior in adolescence: Secondary analysis of cross-sectional data from a statewide youth survey

Table S1. Comparison of Student Demographics: KIP 2021 and Kentucky Department of Education Enrollment 2020-2021

	KIP 2021		KY Total Enrollment 2020-2021[b]		KY Enrollment w/o Jefferson County 2020-2021[c]	
Characteristics	*n*	(%)	*n*	(%)	*n*	(%)
Overall	93,812	(100)	217,566	(100)	185,515	(100)
School Grade						
6	25,552	(27.2)	54,479	(25.0)	46,490	(25.1)
8	26,712	(28.5)	56,558	(26.0)	48,281	(26.0)
10	23,632	(25.2)	56,352	(25.9)	47,633	(25.7)
12	17,916	(19.1)	50,177	(23.1)	43,111	(23.2)
Gender Identity[a]						
Female	42,065	(44.8)	106,035	(48.7)	90,116	(48.6)
Male	46,052	(49.1)	111,531	(51.3)	95,399	(51.4)
Questioning/unsure	1,524	(1.6)				
Not listed	2,103	(2.2)				
Prefer not to say	1,756	(1.9)				
Race-Ethnicity[a]						
Non-Hispanic White	66,829	(71.2)	162,853	(74.9)	149,495	(80.6)
Black	5,920	(6.3)	24,480	(11.3)	12,356	(6.7)
Hispanic or Latino	8,265	(8.8)	16,650	(7.7)	12,898	(7.0)
Asian	1,227	(1.4)	3,867	(1.8)	2,599	(1.4)
Native Hawaiian or Pacific Islander	148	(0.2)	298	(0.1)	253	(0.1)
American Indian or Alaska Native	918	(1.0)	293	(0.1)	257	(0.1)
Multiracial	5,619	(6.0)	9,125	(4.2)	7,657	(4.1)
Other	1,743	(1.9)				
Rural						
Yes	52,087	(55.5)	107,506	(49.4)	107,506	(58.0)
No	41,725	(44.5)	110,060	(50.6)	77,895	(42.0)

[a]Overall characteristics may not add to 100% due to missing data; [b]Total enrollment for Grades 6, 8, 10, and 12 with Jefferson County School District; [c]Total enrollment for Grades 6, 8, 10, and 12 without Jefferson County School District.

Table S2. Characteristics of middle school students

	Overall		SPD		Suicide Ideation		Suicide Attempt	
Characteristics	*n*	(%)	*n*	(%)	*n*	(%)	*n*	(%)
Overall	52,264	(100)	9,217	(17.6)	6,179	(11.8)	3,876	(7.4)
School Grade								
6	25,552	(48.9)	3,823	(15.0)	2,443	(9.6)	1,669	(6.5)
8	26,712	(51.1)	5,394	(20.2)	3,736	(14.0)	2,207	(8.3)
Gender Identity[a]								
Female	22,866	(43.7)	4,791	(21.0)	3,114	(13.6)	1,974	(8.6)
Male	25,821	(49.4)	2,729	(10.6)	1,733	(6.7)	1,101	(4.3)
Questioning/unsure	993	(1.9)	559	(56.3)	431	(43.4)	241	(24.3)
Not listed	1,220	(2.3)	714	(58.5)	595	(48.8)	353	(28.9)
Prefer not to say	1,181	(2.3)	401	(34.0)	290	(24.6)	195	(16.5)
Sexual Orientation[a]								
Heterosexual	32,689	(62.6)	4,075	(12.5)	2,476	(7.6)	1,459	(4.5)
Gay or lesbian	2,893	(5.5)	1,464	(50.6)	1,218	(42.1)	763	(26.4)
Questioning/unsure	2,768	(5.3)	761	(27.5)	536	(19.4)	306	(11.1)
Not listed	3,717	(7.1)	1,437	(38.7)	1,151	(31.0)	682	(18.4)
Prefer not to say	4,861	(9.3)	834	(17.2)	421	(8.7)	322	(6.6)
Race-Ethnicity[ab]								
NH White (White)	35,439	(67.8)	6,126	(17.3)	3,992	(11.3)	2,290	(6.5)
NH Black (Black)	3,555	(6.8)	534	(15.0)	442	(12.4)	327	(9.2)
Hispanic	4,839	(9.3)	931	(19.2)	648	(13.4)	492	(10.2)
NH Asian/NH/PI (Asian/Pacific Islander)	700	(1.3)	112	(16.0)	74	(10.6)	41	(5.9)
NH AI/AN (Native American)	650	(1.2)	137	(21.1)	85	(13.1)	70	(10.8)
NH Other (Other)	1,348	(2.6)	268	(19.9)	182	(13.5)	133	(9.9)
NH Multiracial (Multiracial)	3,242	(6.2)	799	(24.7)	572	(17.6)	378	(11.7)
Rural								
Yes	28,925	(55.3)	5,322	(18.4)	3,541	(12.2)	2,233	(7.7)
No	23,339	(44.7)	3,895	(16.7)	2,638	(11.3)	1,643	(7.0)
Perceived Racial Discrimination[a]								
Yes	2,557	(4.9)	1,018	(39.8)	763	(29.8)	529	(20.7)
No/Not sure	43,225	(82.7)	7,178	(16.6)	4,778	(11.1)	2,818	(6.5)

[a]Overall characteristics may not add to 100% due to missing data; [b]NH= Non-Hispanic; NH/PI=Native Hawaiian or Pacific Islander; AI/AN=American Indian or Alaska Native

Table S3. Characteristics of high school students

	Overall		SPD		Suicide Ideation		Suicide Attempt	
Characteristics	*n*	(%)	*n*	(%)	*n*	(%)	*n*	(%)
Overall	41,548	(100)	10,093	(24.3)	6,345	(15.3)	3,048	(7.3)
School Grade								
10	23,632	(56.9)	5,715	(24.2)	3,694	(15.6)	1,870	(7.9)
12	17,916	(43.1)	4,378	(24.4)	2,651	(14.8)	1,178	(6.6)
Gender Identity[a]								
Female	19,199	(46.2)	5,792	(30.2)	3,354	(17.5)	1,618	(8.4)
Male	20,231	(48.7)	3,065	(15.1)	2,071	(10.2)	967	(4.8)
Questioning/unsure	531	(1.3)	330	(62.2)	253	(47.7)	112	(21.1)
Not listed	883	(2.1)	596	(67.5)	452	(51.2)	227	(25.7)
Prefer not to say	575	(1.4)	283	(49.2))	196	(34.1)	109	(19.0)
Sexual Orientation[a]								
Heterosexual	30,199	(72.7)	5,461	(18.1)	3,124	(10.3)	1,439	(4.8)
Gay or lesbian	2,551	(6.1)	1,375	(53.9)	1,029	(40.3)	512	(20.1)
Questioning/unsure	1,575	(3.8)	704	(44.7)	495	(31.4)	224	(14.2)
Not listed	2,879	(6.9)	1,527	(53.0)	1,122	(39.0)	514	(17.9)
Prefer not to say	1,686	(4.1)	471	(27.9)	255	(15.1)	136	(8.1)
Race-Ethnicity[ab]								
NH White (White)	31,390	(75.6)	7,637	(24.3)	4,726	(15.1)	2,140	(6.8)
NH Black (Black)	2,365	(5.7)	473	(20.0)	319	(13.5)	177	(7.5)
Hispanic	3,426	(8.3)	822	(24.0)	517	(15.1)	296	(8.6)
NHAsian/NH/PI (Asian/Pacific Islander)	675	(1.6)	139	(20.6)	88	(13.0)	40	(5.9)
NH AI/AN (Native American)	268	(0.7)	63	(23.5)	40	(14.9)	31	(11.6)
NH Other (Other)	395	(1.0)	104	(26.3)	67	(17.0)	44	(11.1)
NH Multiracial (Multiracial)	2,377	(5.7)	719	(30.3)	504	(21.2)	269	(11.3)
Rural								
Yes	23,162	(55.8)	5,760	(24.9)	3,603	(15.6)	1,828	(7.9)
No	18,386	(44.3)	4,333	(23.6)	2,742	(14.9)	1,220	(6.6)
Perceived Racial Discrimination[a]								
Yes	3,018	(7.3)	1,122	(37.2)	802	(26.6)	422	(14.0)
No/Not sure	34,581	(83.2)	8,084	(23.4)	4,992	(14.4)	2,259	(6.5)

[a]Overall characteristics may not add to 100% due to missing data; [b]NH= Non-Hispanic; NH/PI=Native Hawaiian or Pacific Islander; AI/AN=American Indian or Alaska Native

Figure S1. Prevalence of perceived racial discrimination among racial and ethnic minorities stratified by school level

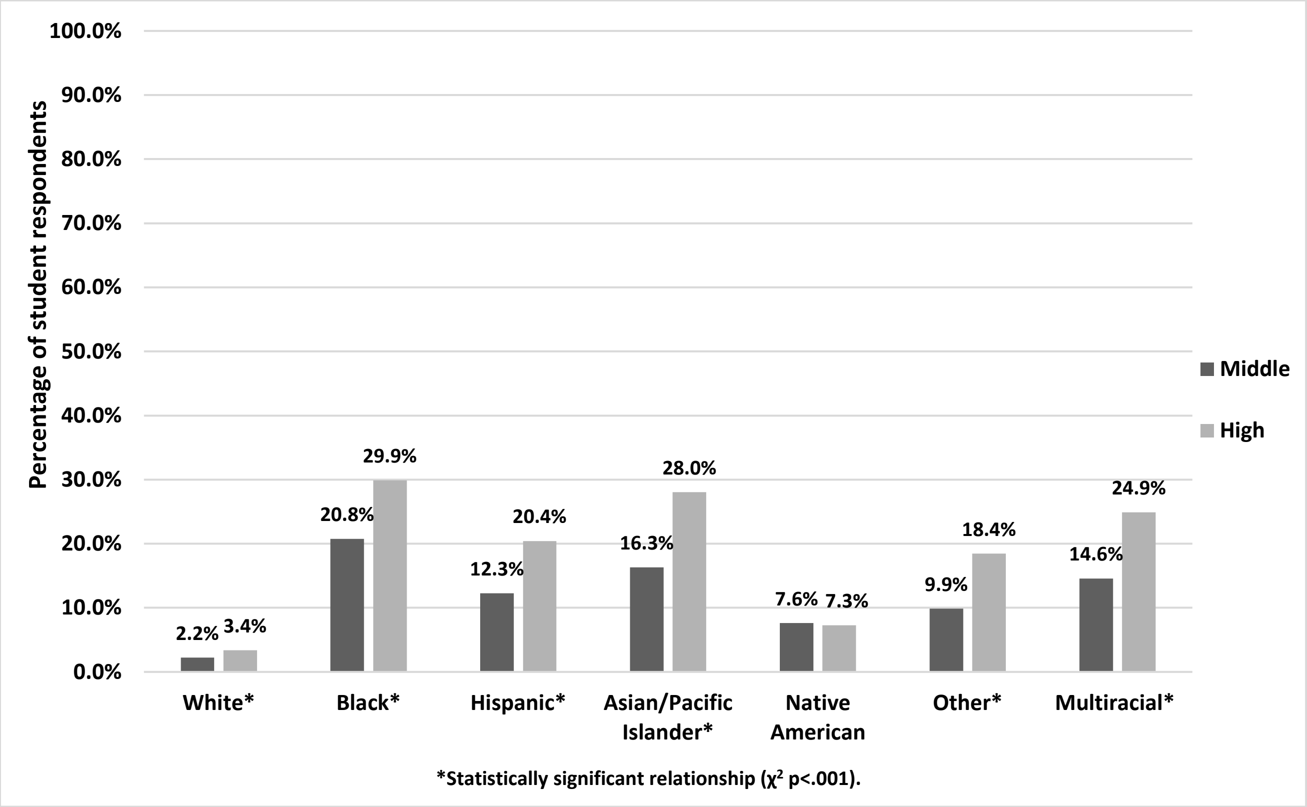

Note: For all racial/ethnic groups besides Native Americans, high schoolers reported significantly higher rates of PRD than middle school students. The following are Cramer's V for each racial/ethnic group with a significant relationship from left to right: 0.04, 0.10, 0.11, 0.14, -0.01, 0.11, 0.13.

Figure S2. Prevalence of SPD and suicidality among racial and ethnic minorities in middle school

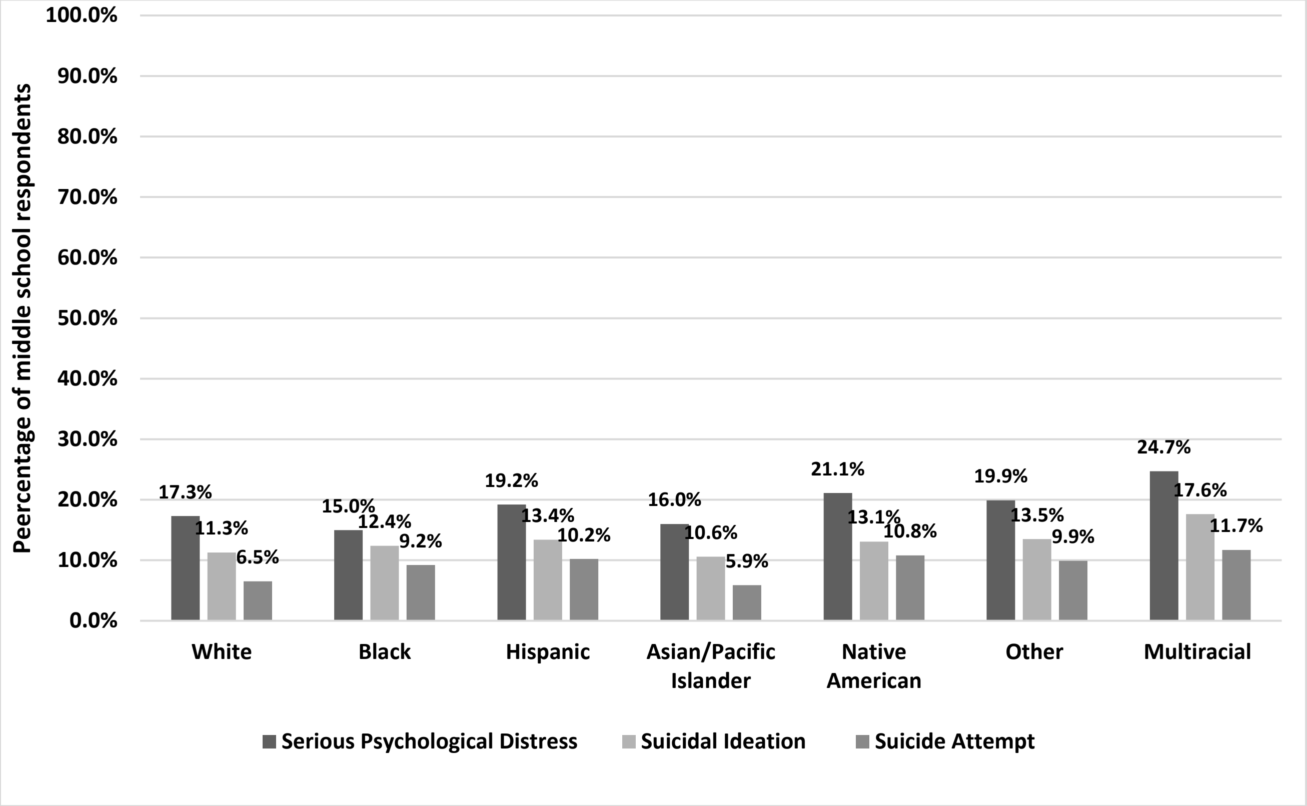

Figure S3. Prevalence of SPD and suicidality among racial and ethnic minorities in high school

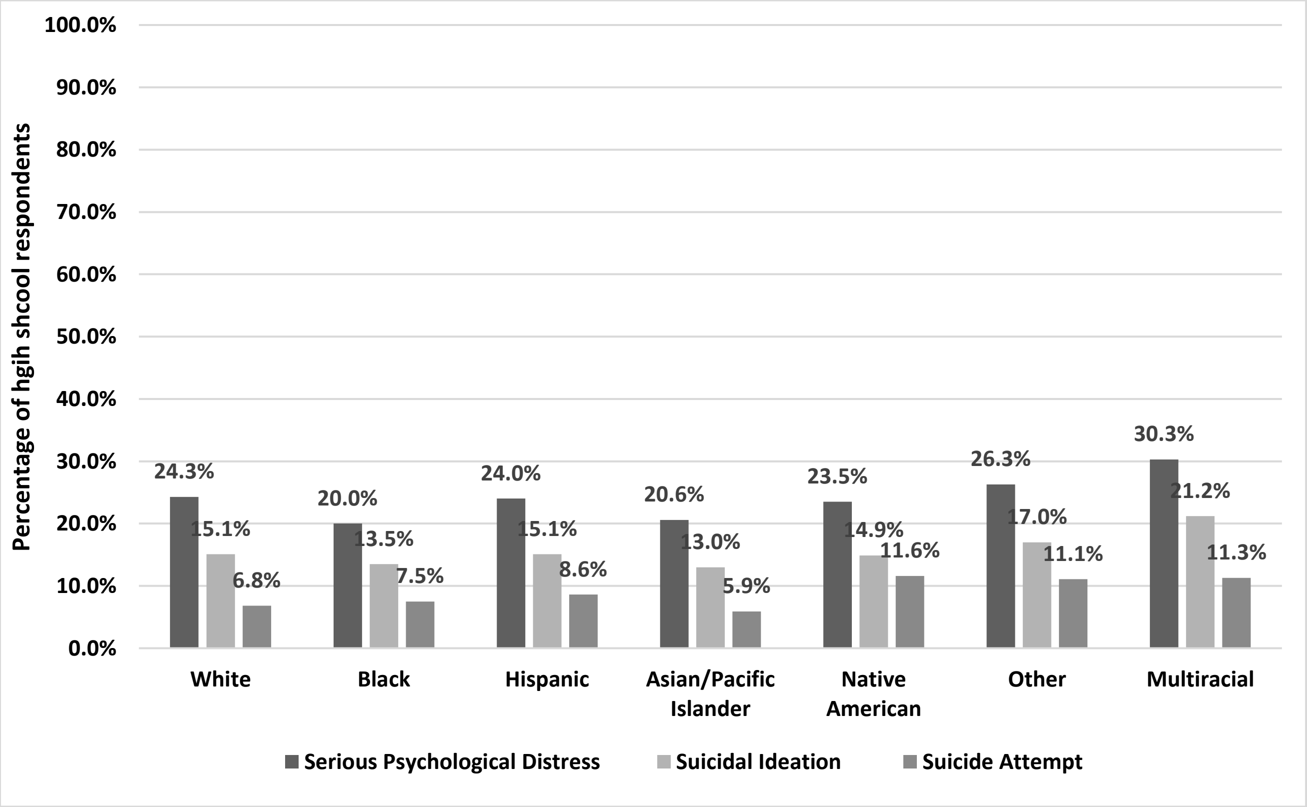

Figure S4. Adjusted odds of SPD and among racial and ethnic minorities stratified by school level

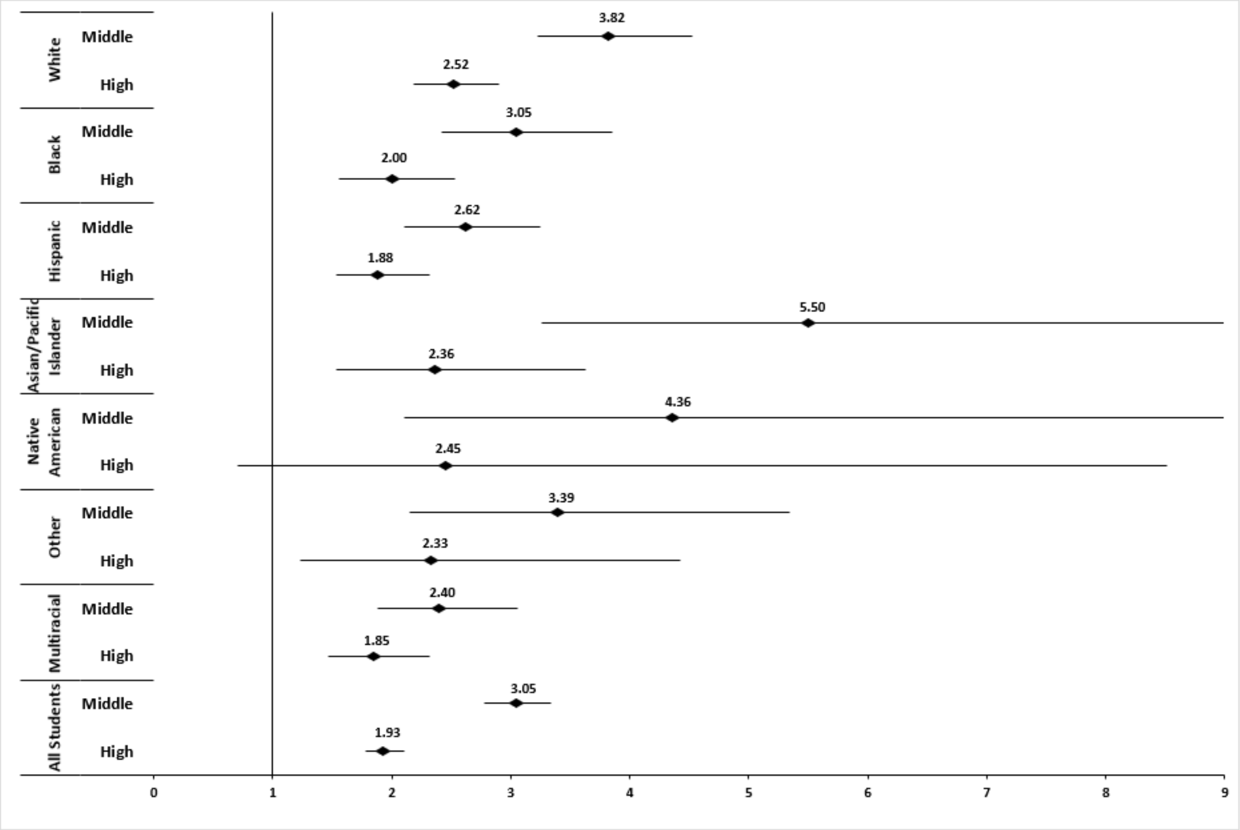

Figure S5. Adjusted odds of suicidal ideation among racial and ethnic minorities stratified by school level

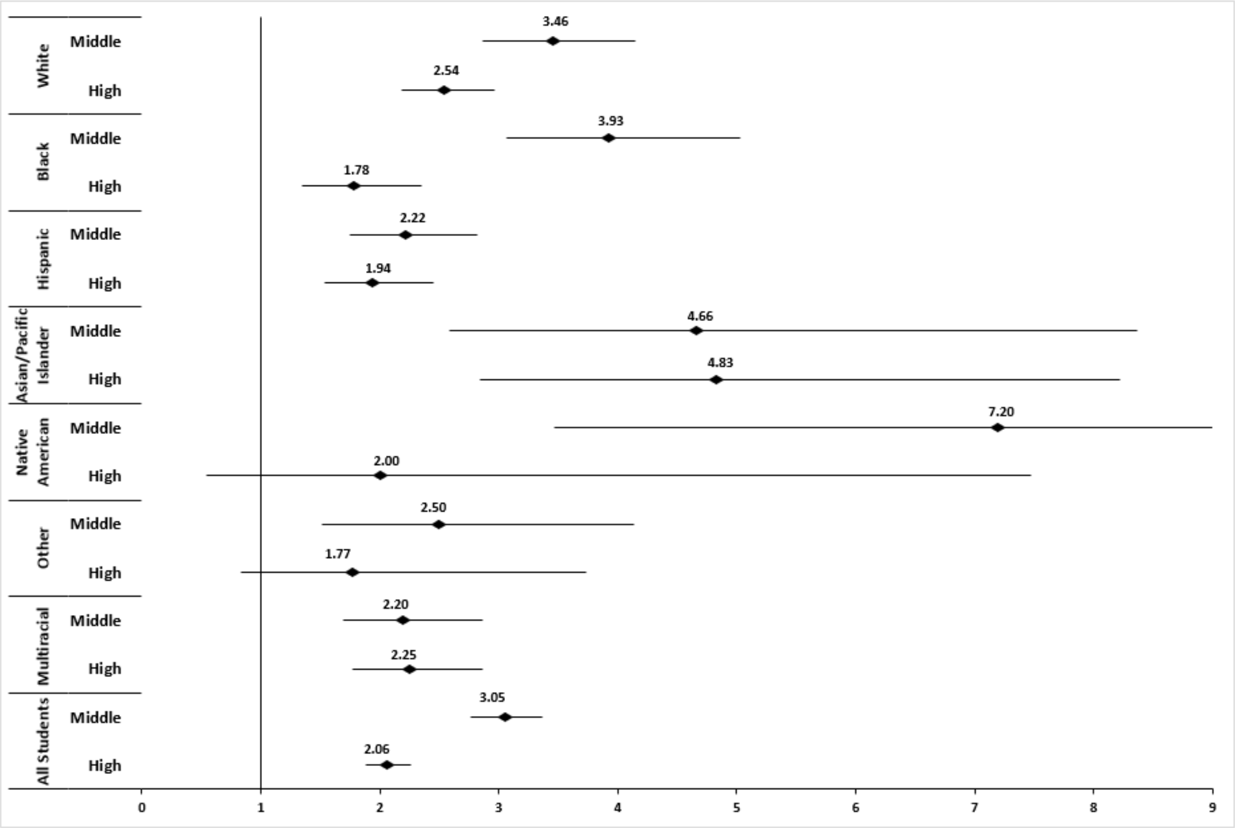

Figure S6. Adjusted odds of suicide attempt among racial and ethnic minorities stratified by school level

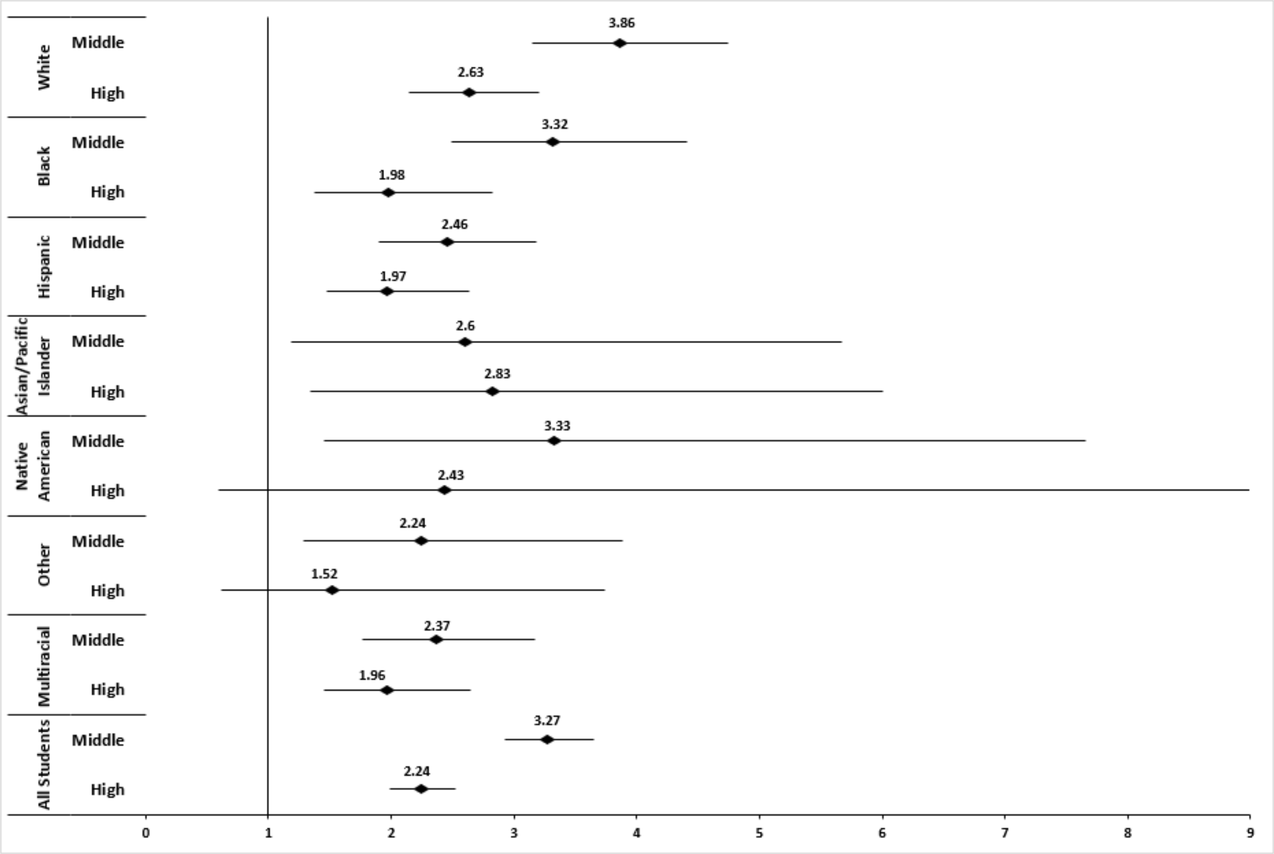

Supplement: Supplementary file 1 [file healthcare-12-01011-s001.zip › healthcare-2961710-supplementary.pdf]
